# Single-file transport of water through membrane channels

**DOI:** 10.1039/c8fd00122g

**Published:** 2018-09-28

**Authors:** Andreas Horner, Peter Pohl

**Affiliations:** Johannes Kepler University Linz, Institute of Biophysics, Gruberstr. 40, 4020 Linz, Austria

## Abstract

Water at interfaces governs many processes on the molecular scale from electrochemical and enzymatic reactions to protein folding. Here we focus on water transport through proteinaceous pores that are so narrow that the water molecules cannot overtake each other in the pore. After a short introduction into the single-file transport theory, we analyze experiments in which the unitary water permeability, *p*_f_, of water channel proteins (aquaporins, AQPs), potassium channels (KcsA), and antibiotics (gramicidin-A derivatives) has been obtained. A short outline of the underlying methods (scanning electrochemical microscopy, fluorescence correlation spectroscopy, measurements of vesicle light scattering) is also provided. We conclude that *p*_f_ increases exponentially with a decreasing number *N*_H_ of hydrogen bond donating or accepting residues in the channel wall. The variance in *N*_H_ is responsible for a more than hundredfold change in *p*_f_. The dehydration penalty at the channel mouth has a smaller effect on *p*_f_. The intricate link between *p*_f_ and the Gibbs activation energy barrier, $∆G_{t}^{‡}$ for water flow suggests that conformational transitions of water channels act as a third determinant of *p*_f_.

## 1 Water at interfaces

Interfacial water is of crucial importance in many technological, environmental and biological processes. For example, it is ubiquitously present as a thin film at hydrophilic surfaces in humid air, it governs electrochemical reactions on liquid–solid interfaces or enzymatic reactions in the active centers of proteins, it is an important determinant for protein folding and it regulates the uptake and release of gases at the liquid–vapor interface of oceans and clouds.^1,2^ Substrates influence the structure and properties of adjacent water molecules. Examples are provided by one-dimensional clusters formed at metal surfaces such as Cu(110)^3^ and oscillatory density profiles within a distance of 10 Å near solid-aqueous interfaces.^4^

One of the manifold functional consequences of the altered physicochemical properties of water is illustrated by the decrease in the dielectric constant from 78 to roughly four on top of a solid surface (mica).^5^ A similar observation has been made in the immediate vicinity of biological membranes.^6^ Yet another example is provided by the water assisted movement of protons at solid surfaces^7,8^ or along membranes.^9–11^ In contrast to hydrophilic substances, water reduces the number of hydrogen bonds adjacent to extended hydrophobic substrates.^12^ As a result, the first layer of water molecules retracts from the surface – adopting a vapor-like structure.^13^ A non-hydrogen bonded OH group (dangling OH) emerges that points toward the hydrophobic surface.^14^ In neuroscience such liquid–vapor oscillations were hypothesized to occur in hydrophobic segments of membrane channels.^15^ This so-called hydrophobic gating may be responsible for keeping the channel functionally closed in the absence of the neurotransmitter. Otherwise, the vapor-filled constriction zone of the channel would be wide enough to allow the passage of both water molecules and ions.

Water structuring on the interface may be expected to have implications on water mobility. Assessment by NMR spectroscopy revealed that water reorientation and translation are 3–5 times slower than in the bulk.^16,17^ The level of mobility reduction is highly heterogeneous at the surface of lipid bilayers^18^ and proteinaceous structures. That is, the dynamics of water reproduce the chemical features of amino acids.^1^ The differences in water motion usually decay within five water layers from the surface.^19^ Protein crowding may prevent the decay from happening. It was reported to leave the number of H-bonds practically unchanged while affecting the H-bond dynamics.^20^ Very little is known about their effect on fluid flow through narrow channels.

## 2 Hydrodynamics

It is clear that the macroscopic laws of hydrodynamics (*e.g.* Hagen-Poiseuille’s law) do not apply to narrow biological channels.^1^ That is, the channel length and diameter can no longer act as major determinants of fluid flow. Moreover, it is impossible to imagine that the outermost water layer adheres to the channel wall, since otherwise there would be no flow across pores that are only one water molecule wide. Already tens of nanometers wide, carbon nanotubes show an enhanced permeability that can only be described by Poiseuille flow with slippage inside the nanotube.^21^ In the extreme case of biological channels specialized in water transport (aquaporins, AQPs), slippage may be perfect, *i.e.* the water molecules may retain a mobility that is close to the mobility of bulk water.^22^

Contributions aiming at a theoretical description of the hydrodynamics in very narrow channels are scarce. Existing ones represent the channels as cylindrical pipes and ignore the interaction of the permeating water molecules with the channel wall. Commonly, partial water dehydration at the channel entrance is assumed to be the major energetic barrier for facilitated water transport.^23^ In contrast, water movement through the single-file region of the channel is treated as if it was not offering any resistance.^24^ In consequence, the predictive power of such models for water flow through biological channels is limited. For carbon nanotubes the situation is different. Here, the dehydration penalty may be rate determining. Existing models envision water flow as (i) a collective motion of the entire single-file water column in a random walk fashion^25^ or as (ii) the movement of a vacancy in the direction opposite to that of water movement.^26^ An overview of single-file water in artificial nanopores has been given previously,^27^ allowing this review to focus on biological channels.

Here we reproduce a short outline of the most influential model of the past decades that has been proposed by Alan Finkelstein^28^ long before water selective channel proteins, *i.e.* AQPs, were discovered. The model envisions the work *W* required to move *N* water molecules through the channel to be equal to:

(1) $$W=\nu_{w}N∆\Pi$$

where Δ*Π* and *ν*_w_ are the osmotic pressure difference and the volume of one water molecule. The model also postulates that W is proportional to channel length *L*:

(2) $$W=F_{\Pi }L$$

where *F*_Π_ denotes the osmotic force. Strictly speaking, this assumption is correct for hard spheres rolling through a pipe. The situation in water channeling proteins may be very different. Now, available structural information indicates that even single-file channels may have constriction zones which impose more resistance to water flow than wider regions.^29^ In addition, pore lining hydrogen bond donating and receiving residues are not evenly distributed.^30^ That is, the permeating water molecules may be bound in one place, but they may not find interaction partners in others. Consequently, most of the resistance to water flow could be generated in one spot. Lengthening the channel beyond that region would result in a negligible increment of *W*.

It is illuminating to follow Finkelstein’s derivation of unitary water channel permeability, *p*_f_, till the end, even though we disagree with eqn (2) on the grounds that there is no experimental evidence for *W* to be proportional to *L*. We should also keep in mind that eqn (2) implies the dehydration penalty be much smaller than *W*.

Expressing *F*_Π_ from eqn (1) and (2) and setting it equal to the frictional force *F*_γ_:

(3) $$F_{\gamma }=N_{\gamma \nu }$$

allows the velocity *ν* of the water molecules in the pore to be obtained:

(4) $$v=\frac{v_{w}∆\Pi }{L_{\gamma }}$$

where γ is the friction coefficient. Substituting Δ*Π* for *kT*Δ*n*_s_,γ for *kT/D*_1_ and using the definition of the unitary channel permeability *p*_f_ = *Φ*/Δ*n_s_*, where the flux *Φ* = *Nν/L*, we find:

(5) $$p_{\text{f}}=\frac{v_{w}D_{1}N}{L^{2}}.$$

**Fig. 1:**
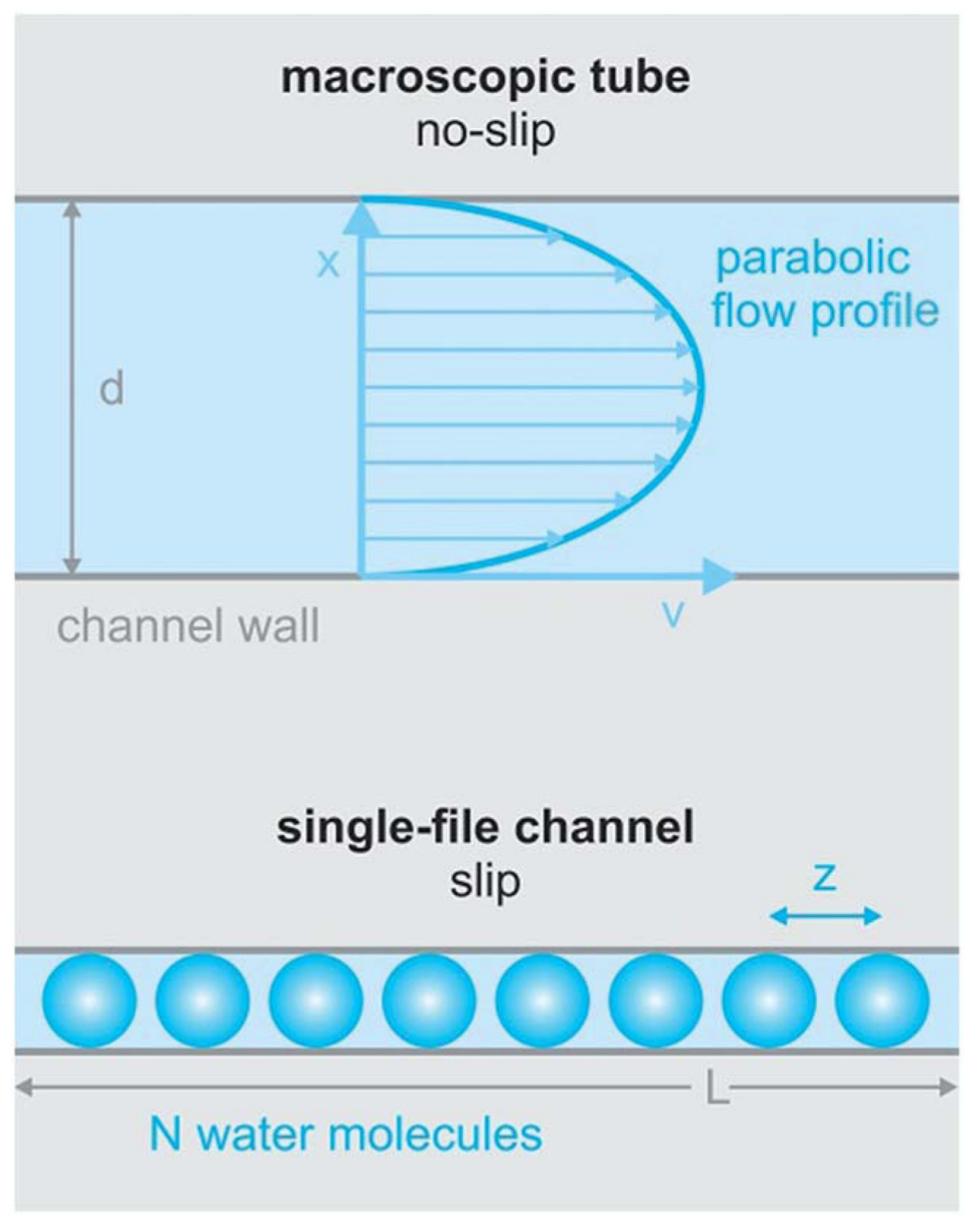
The difference between macroscopic and sub-nanometer hydrodynamics. Hagen–Poiseuille’s law envisions a parabolic streaming profile through macroscopic tubes due to a no-slip condition at the channel wall. In contrast, there cannot be a parabolic streaming profile in single-file flow (eight water molecules are drawn in blue – not to scale). Thus, the no-slip condition is not obeyed in narrow biological channels. *L* denotes the length of the channel, *N* denotes the number of single-file water molecules and *z* denotes the distance between adjacent water molecules.

Δ*n_s_* is the transmembrane concentration difference of the impermeant osmolyte and *D*_1_is the diffusion coefficient of a single-water molecule in the pore. Substituting *N* for *L/z*, where *z* is the distance between two water molecules (Fig. 1) transforms eqn (5) into:

(6) $$p_{\text{f}}=\frac{v_{w}D_{1}}{zL}.$$

Eqn (6) states that *p*_f_ is inversely proportional to *L*. This important result is a direct consequence of the assumption *W ~ L* made by eqn (2). The problem with eqn (6) is that *D*_1_ is a purely theoretical parameter. There is no isolated singlewater molecule in the pore. Water moves in a column of *N* water molecules. This is thought to reduce its mobility *N* times,^31^ *i.e.* the diffusion coefficient *D*_w_ of a water molecule in the pore is equal to $D_{\text{w}}=\frac{D_{1}}{N}$. Such an observation has been made for hard spheres in a cylindrical pipe. The presence of a constriction zone as well as the availability of hydrogen bond donors and acceptors in the wall of a membrane channel may confound the analysis. That is, the same reservations that have been raised against eqn (2) apply here.

Most interestingly, the two questionable length dependencies (for *D*_w_ and *W*) cancel each other out when substituting *D*_1_ for *D*_w_. Inserting *N* × *z* for *L* transforms eqn (6) into:

(7) $$p_{\text{f}}=\frac{D_{w}v_{w}}{{\text{z}}^{2}}.$$

Eqn (7) has independently been derived to extract *p*_f_ values from molecular dynamics simulations.^25,32^ It shows that *p*_f_ and *D*_w_ are interchangeable parameters for fully occupied water channels. The drawback of eqn (7) is that it does not allow for making any predictions about how fast a given channel will conduct water. That information is hidden behind *D*_w_ and can only be unveiled by an analysis of the interaction between the pore lining residues and the permeating water molecules.

## 3 Water mobilities inside narrow membrane channels vary widely

Eqn (6) predicts very limited variability of *p*_f_ for membrane channels. In the case of homogeneous interactions with the channel wall, *i.e.* for an invariant *D*_1_,*p*_f_ may vary no more than two times between different channels. The simple reason is that known single-file regions are at least four water molecules long (potassium channels) and accommodate no more than eight water molecules (AQPs).

The attempt to verify the prediction (eqn (6)) experimentally faced large obstacles. Progress in determining the *p*_f_ value of water channeling proteins has been slow due to large experimental difficulties. In contrast to ion channel abundance, which can simply be measured by observing conductivity steps when a constant voltage is applied across a membrane patch, water channel density in the membrane patch is difficult to assess. The background conductivity of lipid bilayers to water is much too large to enable single channel recordings of AQPs. Thus, AQP counting often relies on immunohistochemistry or other biochemical assays with limited accuracy. For example, determining the water channel concentration *via* the bicinchoninic acid assay (BCA assay) depends on a standard curve from a known, standard protein. There is a good chance that the membrane protein does not interact with the dye in a similar fashion to the standard water soluble protein (commonly bovine serum albumin, BSA) because the relative amount of hydrophobic amino acids insoluble in membrane proteins must be different. Since the BCA reaction is influenced by cysteine, tyrosine, and tryptophan residues, the membrane protein concentration may be overestimated by a large margin.

In addition to counting problems, unstirred layers, *i.e.* stagnant water layers, in the immediate membrane vicinity, may hamper the results. The simple reason is that upon entering the hyperosmotic solution water dilutes the osmolyte within these unstirred layers and thus diminishes the driving force for the transmembrane water flux.^33,34^ Finally, other methodological problems that arise when (i) extracting water efflux from time dependent changes of the vesicle or cell volume and (ii) using light scattering to continuously measure the volume of a shrinking particle, have contributed to the great variability of reported *p*_f_ values.

**Fig. 2:**
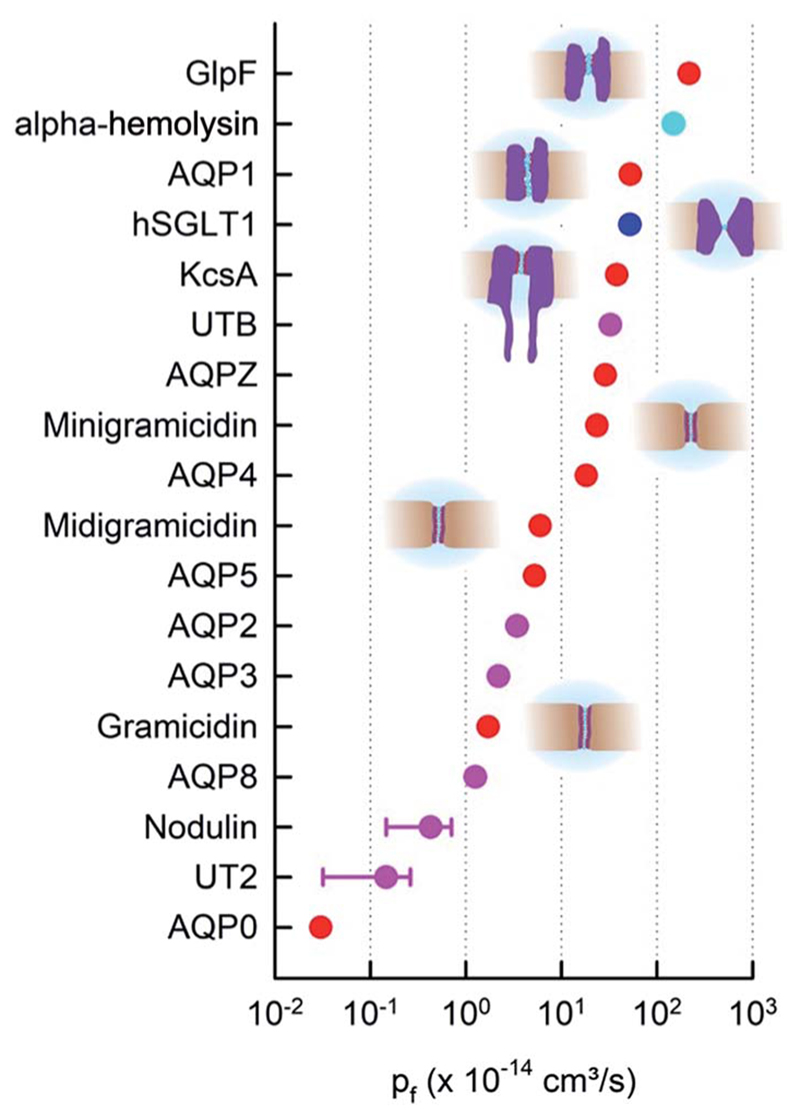
The *p*_f_ values of membrane channels and transporters. The red and pink dots indicate pores with a known single-file region. Whether the sodium-glucose cotransporter SGLT1 (blue) has a single-file region is not entirely clear. The beta-barrel α-hemolysin (cyan) accommodates multiple water molecules in its cross-section.^35^ Moreover, red and blue dots indicate quantitative *p*_f_ measurements in terms of protein counting. hSGLT1,^36^ KcsA, GlpF, AQP1, AQPZ,^22^ AQP4,^37^ and AQP5^38^ were counted with fluorescence correlation spectroscopy (FCS). The bacterial cation selective gramicidin channels^39^ were measured electrically and AQP0 (ref. 40) with electron microscopy (EM). AQP8 (ref. 41) is also permeable to ammonia and urea. *Vice versa*, urea transporters Ut-B and UT-2 are permeable to water.^42^

The difference in data quality is reflected in the color code of Fig. 2. The *p*_f_ values of single-file channels that have been obtained (i) by a direct count of the reconstituted channels and (ii) by accounting for unstirred layer effects are indicated in red. Data that (i) are based on a biochemical or immunohistochemical estimate of the actual protein abundance or (ii) may have suffered from unstirred layers or other methodological problems enter Fig. 2 in pink.

In contrast to its expected small variability (eqn (6)), the experimental *p*_f_ values of single-file channels, and thus the intraluminal water mobilities, vary over four orders of magnitude (Fig. 2). The most efficient water channel seems to be the aqua-glycerol facilitator GlpF from *E. coli*.^22^ Water retains bulk-like mobility within the channel. *D*_w_ amounts to about 5 × 10^−5^ cm^2^ s^−1^ according to eqn (6). The slowest water movement has been observed for AQP0 (ref. 40), also called the major intrinsic protein of the lens. The water mobility drops four orders of magnitude below the bulk mobility, as indicated by *D*_w_ 4 × 10^−9^ cm^2^ s^−1^. We conclude that the variation in *L* cannot explain the variability in *p*_f_ (and *D*_w_).

Most interestingly, water traverses both AQP0 and GlpF in single-file.^43,44^ That is, mechanisms must exist that regulate *D*_w_ to adopt such different values, while keeping *L* nearly constant. As outlined below, the availability of hydrogen bond donating or receiving residues in the channel wall is one of the major determinants of water mobility,^22^ the presence of charged residues at the channel mouth is another.^37^ These factors seem to be more important than even an increased channel diameter. For example, the lumen of α-hemolysin is much wider than the diameter of a single water molecule, *i.e.* water molecules and ions may overtake each other in the channel, and yet, its *p*_f_ value is smaller than that of GlpF (Fig. 2). In addition to interactions between permeating water molecules and water pathway lining residues, there may be other factors – like channel gating – that add to the variability in *p*_f_.

## 4 Length dependence of *p*_f_

Based on Finkelstein’s theory, *p*_f_ is widely assumed to linearly decrease with *L* (eqn (6)). However, experimental evidence in support of the postulate is scarce. As outlined above, linearity may not be expected in the case of an inhomogeneous channel that by virtue of a constriction zone may impose a larger barrier to water permeation in one spot than in the remainder of the channel. However, it is also not clear whether the postulate works for a homogeneous channel: the water molecules in the channel are likely to be hydrogen-bonded to residues in the channel wall. These hydrogen bonds should all break at once in order to allow the advancement of the water chain as a whole. The probability of such an event cannot generally be expected to depend linearly on the total number of water molecules in the chain.

**Fig. 3:**
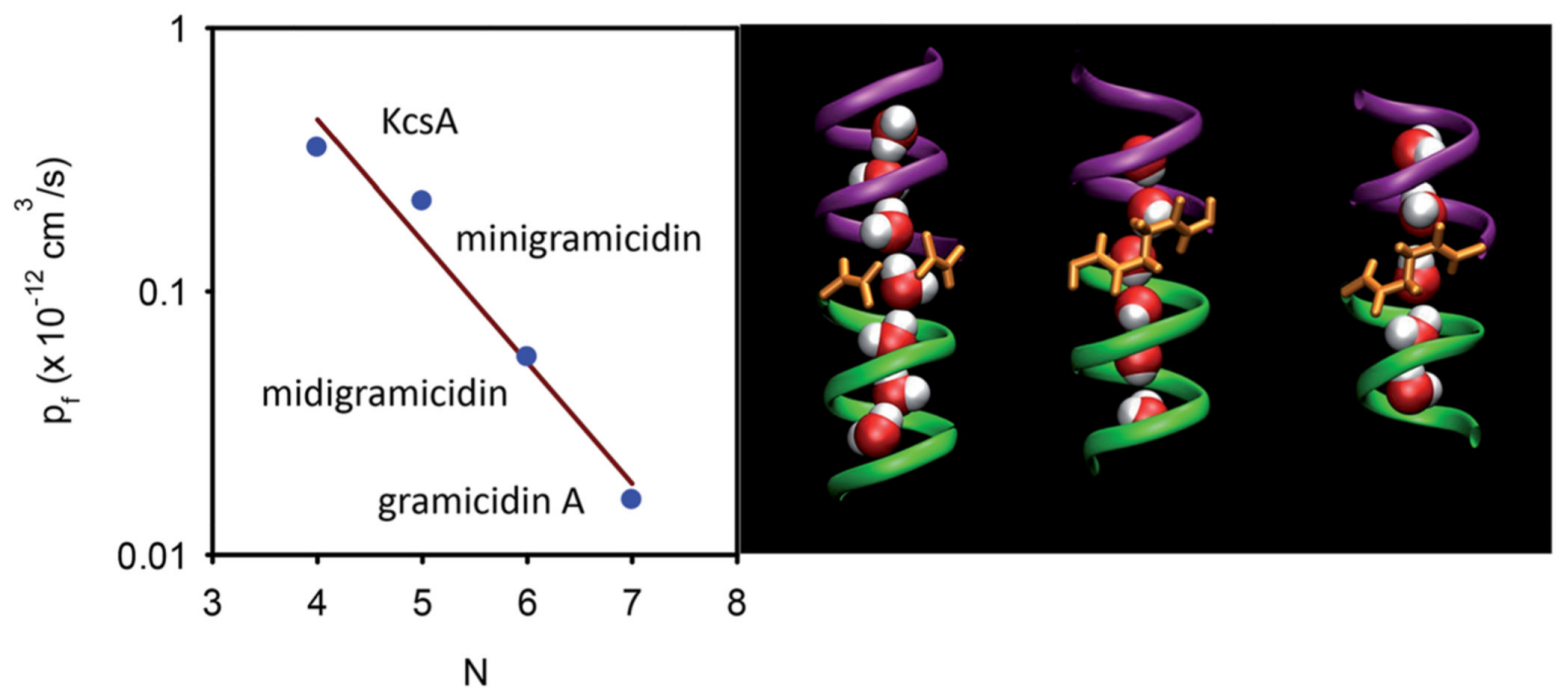
Dependence of *p*_f_ on the length of the single-file. (A) The number *N* of water molecules that line up in a single-file configuration is a crucial determinant of *p*_f_. For the KcsA experiments, see section 6. (B) The length dependence is derived by exploiting three different gramicidin channels (from left to right): wild-type gramicidin A, midigramicidin, and minigramicidin, in addition to the bacterial potassium channel KcsA. The two gramicidin monomers are covalently linked together in the shorter channel versions. The right panel is taken from Saparov *et al., Phys. Rev. Lett.*, 2006.^39^

To test this prediction, we designed homogeneous peptide channels of different lengths.^39^ Gramicidin A served as a template. The wild-type channel is a dimer that consists of two penta-deca peptides which each span only one membrane leaflet. We shortened either one or both monomers in such a dimer and covalently linked the two halves to form midigramicidin and minigramicidin (Fig. 3B). To this end we had channels that accommodated seven,^45^ six^39^ and five^39^ water molecules. They spontaneously inserted into planar lipid bilayers as was indicated by their ion conducting activity, which also served to count the channels. That is, we first measured single channel conductance *g* in separate experiments and assumed that the total electrical conductivity *G* of reconstituted bilayers with many thousands of such channels was equal to *g* multiplied by the number *n* of reconstituted channels: *G* = *N* × *g*.

Both *G* and the integral water permeability *P*_f_ of these membranes were assessed simultaneously. The *P*_f_ measurements were performed by exploiting scanning electrochemical microscopy (SEM). The method itself is outlined in more detail in the next section. In brief, a volume flux is induced by imposing an osmotic gradient across the planar bilayer and the steady-state solute concentration change in the immediate membrane vicinity is recorded by an ion-selective microelectrode in a spatially resolved manner.^34^ *p*_f_ can be derived by plotting *P*_f_ as a function of *G*:

(8) $$P_{\text{f}}=\frac{np_{f}}{A}=\frac{p_{f}}{Ag}G$$

where *A* is the membrane area.

In contrast to theoretical predictions (eqn (6)), *p*_f_ decreases exponentially with *N* (or *L* since *L* = *Nz*) (Fig. 3A).^39^ This is a very important result because it (i) allows mechanistic insight and (ii) has important methodological implications:

(i) The interactions of water molecules in a water chain with residues in the channel wall cannot be represented as a linear superposition of frictional forces, *i.e.* it is not reflected by a model in which hard spheres roll through a pipe. Instead, the concerted formation or breakage of multiple hydrogen bonds between the water chain and channel wall could be reconciled with the exponential dependence of *p*_f_ on *N*.
(ii) Measurements of the ratio of *p*_f_ and the diffusional permeability *p*_d_ are unlikely to reveal *N*, since both parameters have been derived assuming that the energy for moving a water molecule across the channel linearly depends on *L* (eqn (2)).

*p*_d_ is measured in the absence of an osmotic gradient. That is, a tracer (e.g. D_2_O or THO) is added to one compartment and its appearance is monitored on the other side of the membrane. The integral diffusive permeability *P*_d_ of a membrane is equal to *P*_f_ in the absence of membrane channels.^28^ As soon as water channeling proteins are present, *P*_f_ is always larger than *P*_d_.^28^ The reason is that *p*_f_ > *p*_d_. Finkelstein derived the dependence of *p*_d_ ~ 1/*N* ^2^ in a fashion similar to *p*_f_ ~ 1/*N* (eqn (2)–(6)). We do not reproduce the derivation here, because it rests on the incorrect assumption that eqn (2) is valid. Accordingly, the predicted ratio *p*_f_/*p*_d_ = *N* was never confirmed experimentally. Finkelstein himself reports *p*_f_/*p*_d_ ≈ 5 for gramicidin A,^46^ while molecular dynamics simulations show seven molecules in the pore^39^ (Fig. 3B). Mathai *et al.* report *p*_f_/*p*_d_ ≈ 13 for AQP1,^47^ instead of about eight molecules that according to AQP1’s structure may be placed into the single-file region.^29^

## 5 Experimental approaches for determining *p*_f_

Two principally different approaches for membrane water permeability measurements can be distinguished: (a) tracer experiments that are carried out in osmotic equilibrium, and (b) experiments in which an osmotic gradient is established across the membrane. Tracer experiments using NMR spectroscopy^48^ or monitoring the efflux of a radioactive isotope^49^ are very convenient means to determine *P*_d_ in the absence of membrane channels. However they are seldom used for the characterization of water channel permeability – the reason being that there are more convenient and accurate ways of finding *N* than *via* the *p*_f_/*p*_d_ ratio. Consequently, we will not dwell on equilibrium methods, but focus instead on some selected methods for determining *p*_f_.

**Fig. 4:**
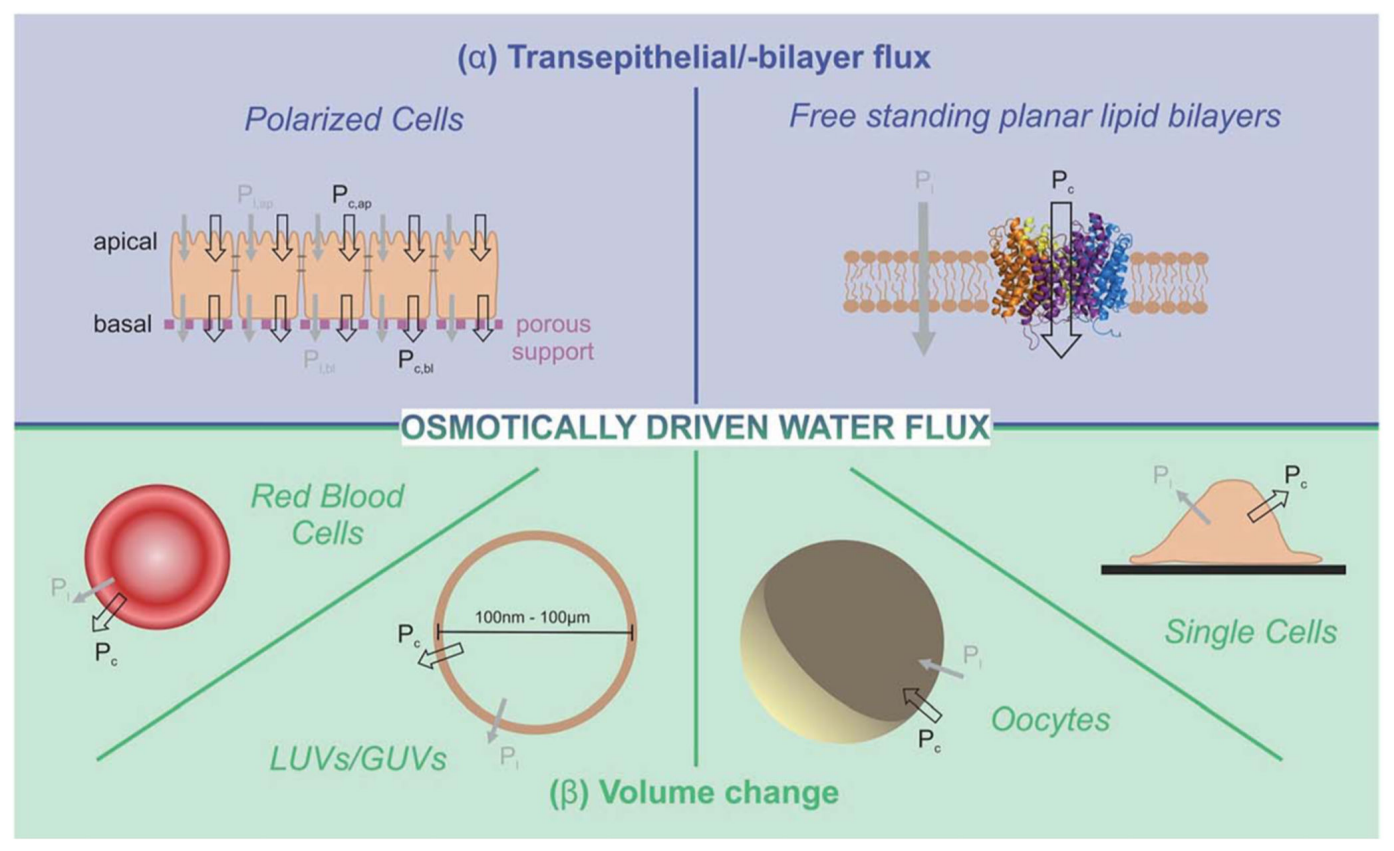
Two different approaches allow estimations of integral osmotic water permeability *P*_f_: (α) continuous flux experiments through a confluent cell monolayer or a free standing planar lipid bilayer (blue background), or (β) volume adaptation of cells or vesicles after an osmotic challenge (green background). In both cases the overall permeability *P*_f_ is the sum of the permeabilities *p*_f,l_ and *P*_f,c_ of the lipid matrix and water conducting channels, respectively. The measurement systems include artificial free standing planar lipid bilayers as well as large or giant unilamellar vesicles (LUVs/GUVs) on the one hand and erythrocytes, oocytes, confluent cell monolayers or single cells on the other hand.

**Fig. 5:**
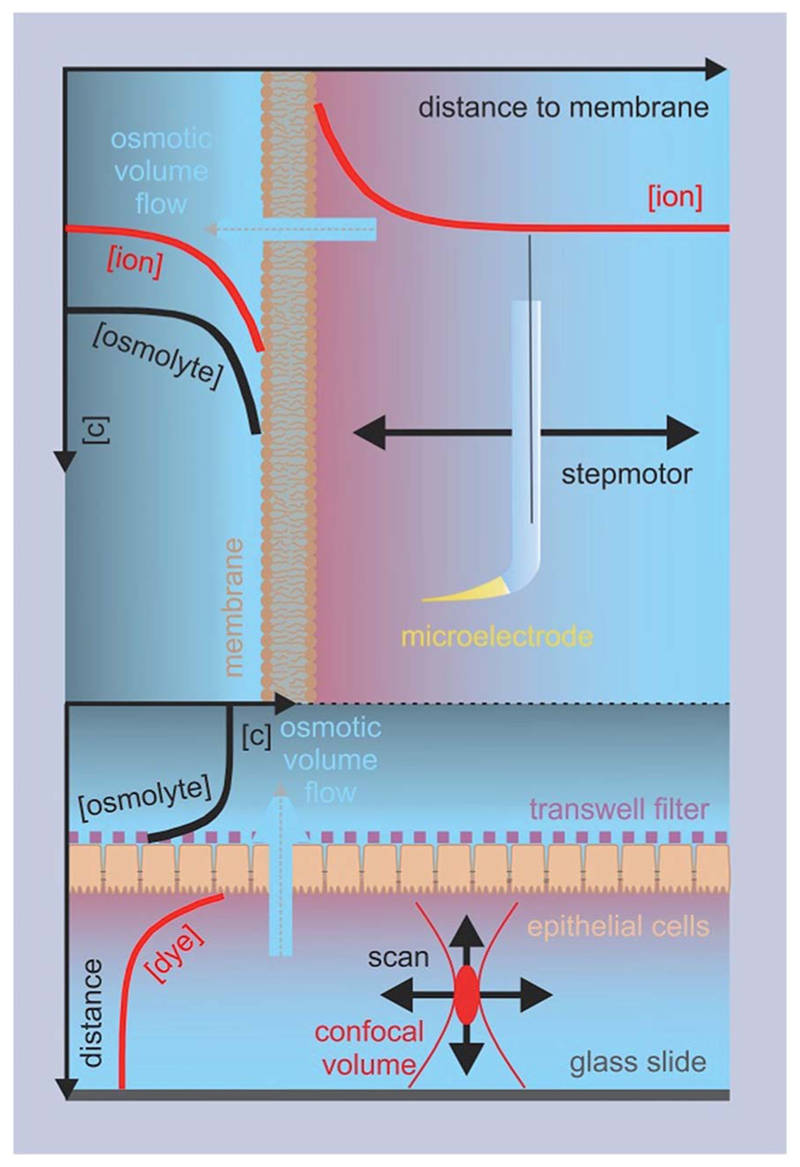
Steady-state or quasi steady-state water flux monitored by means of SEM or FCS, respectively. Top: a hydraulic step motor moves the ion selective microelectrode perpendicular to a free standing planar lipid bilayer. The electrode records the osmotically induced change in ion concentration within the unstirred layer as a function of the distance to the lipid bilayer. The lipid bilayer is folded across an aperture (~100–400 μm diameter) in a Teflon septum that separates the two compartments. Bottom: the focal volume is moved in lateral and/or axial directions through the lower compartment formed by the glass slide and the cell monolayers on a porous filter support. The number of fluorescent dye molecules in the focal volume is derived from fluctuations in the fluorescence intensity.

These methods rely on the presence of a transmembrane osmotic gradient. The assessment of the resulting osmotic flux can be carried out in two principally different ways (Fig. 4): the osmotic flux is monitored either (α) in steady-state or quasi steady-state, or (β) in a kinetic approach. In (α) the volumes of both the donating and receiving compartments are large as compared to the volume of the transmembrane flux per minute, so that the osmotic gradient does not significantly change with time (Fig. 5), whereas in (β) vesicles or cells that enclose a rather limited internal volume are deflated or enlarged (Fig. 7).

### α Transepithelial/-bilayer flux

Osmotic transepithelial or transbilayer water fluxes dilute the solution they enter and concentrate the solution they leave.^50^ Consequently, reporter molecules in one or both compartments can be used to assess *P*_f_.^34^ As such, metal ions are most convenient, since they are usually present in the surrounding solutions. Scanning ion selective microelectrodes are well suited to record the corresponding concentration profiles in the membrane vicinity. In contrast, FCS requires the addition of aqueous fluorescent dyes (Fig. 5). The sensitivity of both methods greatly depends on the size of the unstirred layer, the osmotic gradient, *P*_f_, and the diffusion constant of the reporter molecule.^34,51^ Hence, bigger molecules, which have a smaller diffusion constant, are characterized by a more pronounced concentration shift at the interface. Thus, a clever selection of fluorescently labeled reporter molecules, like dextrans, antibodies or lipid vesicles, may result in a higher sensitivity of FCS measurements as compared to SEM. Sensitivity can be further enhanced by extending the unstirred layers. For example, a small distance between the epithelial cell monolayer and the glass slide (Fig. 5 (bottom)), ensures that transport through the resulting cleft occurs only by diffusion. Both SEM and FCS allow visualization of the effect of inhibitors or other pharmacologically interesting substances on the water flux in one experiment, *i.e.* on one and the same sample.^36,38,41,52^ In contrast, demonstrating the effect of an inhibitor on the rate of water efflux from vesicles or cells (see below) requires performing two subsequent experiments (i.e. using two different samples): one in the presence and one in the absence of the inhibitor.

SEM was used to measure the water flux and solvent drag through gramicidin derivatives,^39,45,53^ to functionally characterize several AQPs,^41,52,54–56^ the translocation complex SecYEG,^57^ and the potassium channel KcsA,^58^ and to investigate different routes of epithelial water flow.^59^ Magnetic stirrers in both compartments ensure that steady-state is reached within a reasonable amount of time.^60^ Because the volume of the two compartments is large as compared to the flow volume, solute bulk concentrations can be assumed to be constant during the experiment. The steady-state solute concentration distribution in the unstirred layer can be used to determine the velocity *ν*_t_ of water flow across the barrier:

(9) $$C\left(x\right)=C_{s}e^{\left(-v_{t}x/D\right)+\left(ax^{3}/3D\right)}$$

where *x*, *D*, *a* and *C*_s_ denote the distance to the membrane, the diffusion coefficient, the stirring parameter, and the solute concentrations at the interface, respectively.^61^ Subsequently, *ν*_t_ allows the calculation of *P*_f_:

(10) $$P_{f}=\frac{v_{t}}{\chi C_{osm}V_{w}}$$

where χ, *C*_osm_ and *V*_W_ are the osmotic coefficient, the near-membrane osmolyte concentration, and the partial molar volume of water, respectively.

In a similar fashion, fluorophore dilution or up concentration adjacent to the cell monolayer was used to calculate the *p*_f_ value of AQP5 (ref. 38) and the sodium-glucose cotransporter hSGLT1.^36^

## 6 Water flux through K^+^ channels

To corroborate the notion that *p*_f_ exponentially depends on length, we substituted gramicidin for another pore: the bacterial potassium channel KcsA. A further shortened gramicidin channel would otherwise have required a matching decrease of bilayer thickness, which would have resulted in rather unstable planar bilayers. The problem does not arise with KcsA since this membrane channel has a “normal” hydrophobic thickness. Its single-file region is much shorter. It is limited to the selectivity filter, which is only four water molecules long. The remaining space is occupied by a water filled cavity that is wide enough to cause negligible resistance to water flow.

**Fig. 6:**
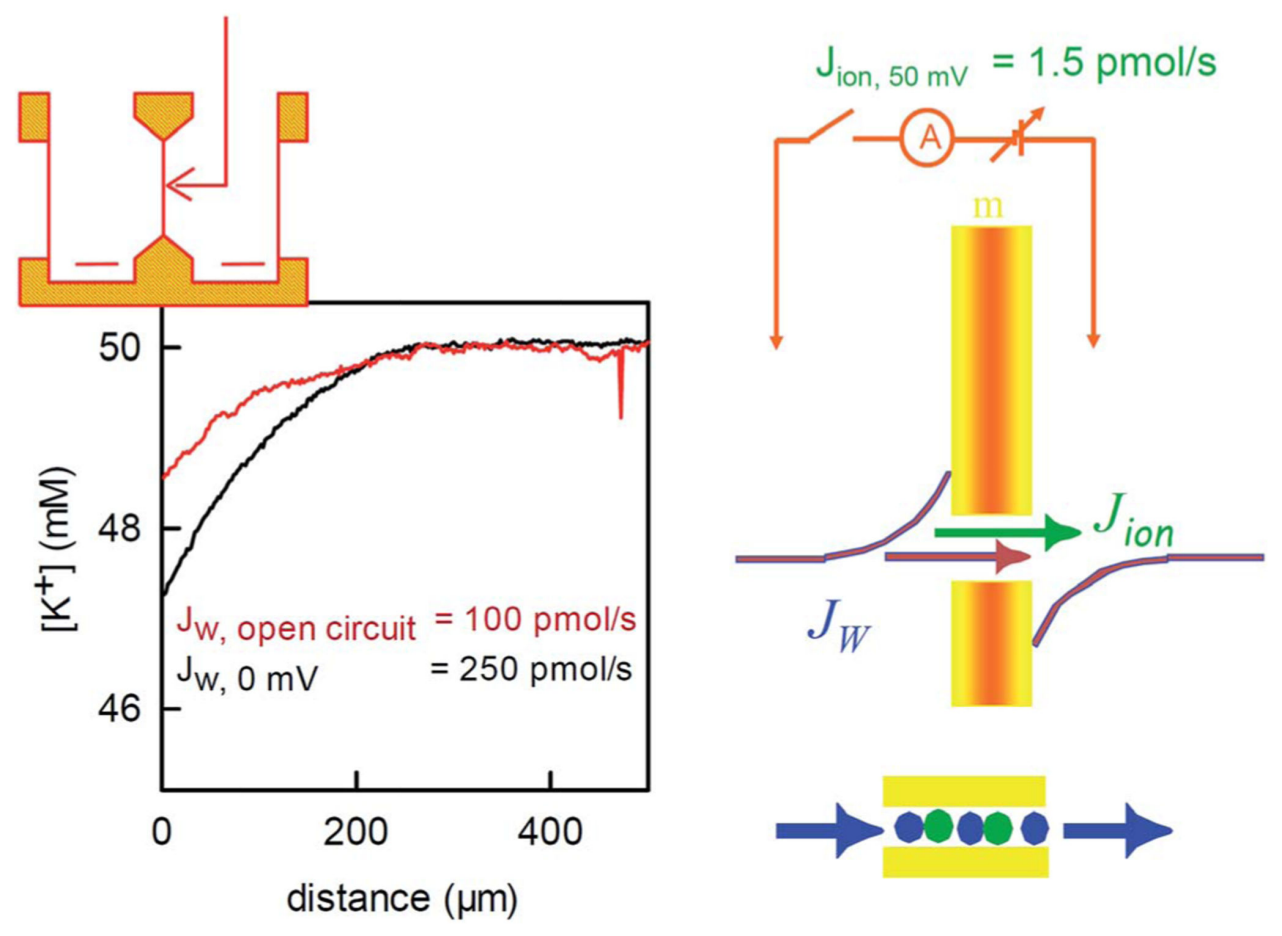
Water flux through the reconstituted bacterial potassium channel KcsA. The potassium channels were reconstituted into planar bilayer lipid membranes. An osmotic gradient (1 M urea) induced a water flux across the membrane (from left to right in the scheme on the top of the left panel). A scanning potassium selective microelectrode in the hyperosmotic compartment recorded the resulting solute dilution (scheme in the middle of the right panel) as a function of the distance to the membrane (graph on the left, the graph has been modified from Saparov *et al.*, PNAS, 2004 (ref. 58) Copyright (2004) National Academy of Sciences). The concentration profile was used to calculate the water flux *J*_w_. Disrupting the electrical connection between the reference electrodes on both sides of the membrane (scheme on the right) decreased *J*_w_. The effect is due to the streaming potential that develops under open circuited conditions. The potential acts to inhibit the ion flow. In turn, the water flow across the channel also stops, since the water molecules cannot overtake the ions in the single-file region (scheme on the right, bottom). The remaining water flow (red concentration profile) passes across the lipid matrix. Water flux across the potassium channels is responsible for the difference between the red and the black profiles. It amounts to 150 pmol s^−1^. The simultaneously measured ion flow is 100 times smaller.

The structure of the selectivity filter shows water molecules separated by ions.^62,63^ Commonly it is believed that the water molecules act as spacers in order to reduce the electrostatic repulsion between the ions.^64^ An alternative model that envisioned a water free selectivity filter^65^ has not been confirmed experimentally.^66^ In contrast, a selectivity filter that contains a reduced amount of ions or is completely free of ions was suggested by streaming potential measurements^67,68^ or by scanning electrochemical microscopy^58^ (Fig. 6), respectively.

The application of an osmotic pressure across a planar bilayer that is reconstituted with potassium channels results in water flow. The flux of potassium ions that are dragged by the water across the channels can be estimated in terms of the short circuit current (Fig. 6). The ion flux was at least a hundred times smaller than the water flow that was simultaneously measured across the channels by scanning potassium selective microelectrodes.^58^ The true ion to water flux ratio is most probably larger, since (i) *J*_w_ is underestimated, because its calculation assumes an impermeable solute, and (ii) the number of dragged ions is overestimated because the Nernst potential for potassium (compare Fig. 6) drives additional ions through the channel in the direction of the water flow. However, even the lower limit of 100 water molecules per one ion indicates that most of the time, the selectivity filter does not contain ions. That is, KcsA behaves like a water channel and can be used to extend the plot of *P*_f_(*N*) beyond the gramicidin data (Fig. 3).

Counting the channels based on their electrical activity resulted in an unreasonably high *p*_f_ value. It corresponded to water molecules that were much more mobile than bulk water molecules.^58^ Channel inactivation provided a possible explanation for the conundrum. That is, 90% of the channels were electrically silent and yet they provided a water pathway.^69^

Evidence was obtained by counting the channels that were reconstituted into LUVs *via* their fluorescence.^69^ The label was attached to an artificially introduced cysteine in the channel. Since LUVs are smaller than the focal volume, we introduced a two-step procedure: first the number of reconstituted vesicles was counted by FCS. Subsequently, the vesicles were dissolved by detergent and the number of channel-containing micelles was counted (see below for a more detailed description). The ratio of the two counts indicated the number of channels per vesicle. The water conducting ability of the reconstituted channels was derived by (i) exposing the proteoliposomes to an osmotic gradient and (ii) continuously monitoring vesicle deflation in terms of the intensity *I*(*t*) of the scattered light. In a subsequent publication we derived (i) an analytical equation that links *P*_f_ to vesicle volume and (ii) an expression that allows calculation of the vesicle volume from *I*(*t*)^22^ (see methodological details in the next section). Together with several point mutations in the channel that prevented both gating^70^ and inactivation,^71^ these technological advancements resulted in the *p*_f_ value that is depicted in Fig. 3. It confirms the hypothesis that shortening the length of the single-file region augments *p*_f_.

### β Cell or vesicle deflation as a means to obtain *p*_f_

Exposure of cells or vesicles at time *t* = 0 to a hyperosmotic solution that contains membrane impermeable solutes of concentration *c*_out_ leads to a *P*_f_ dependent volume shrinkage:

(11) $$\frac{\text{d}V\left(t\right)}{\text{d}t}=AP_{\text{f}}V_{\text{w}}\left(c_{\text{in}}\left(t\right)-c_{\text{out}}\right)$$

(12) $$c_{\text{in}}\left(t\right)=\frac{V_{0}}{V\left(t\right)}c_{\text{in},0}$$

where *V*_0_, *A*, *c*_in,0_ and *c*_out_ denote the vesicle volume at time zero, the time invariant vesicle surface area, the initial osmolyte concentration inside the vesicles, and the osmolyte concentration in the external solution, respectively.

**Fig. 7:**
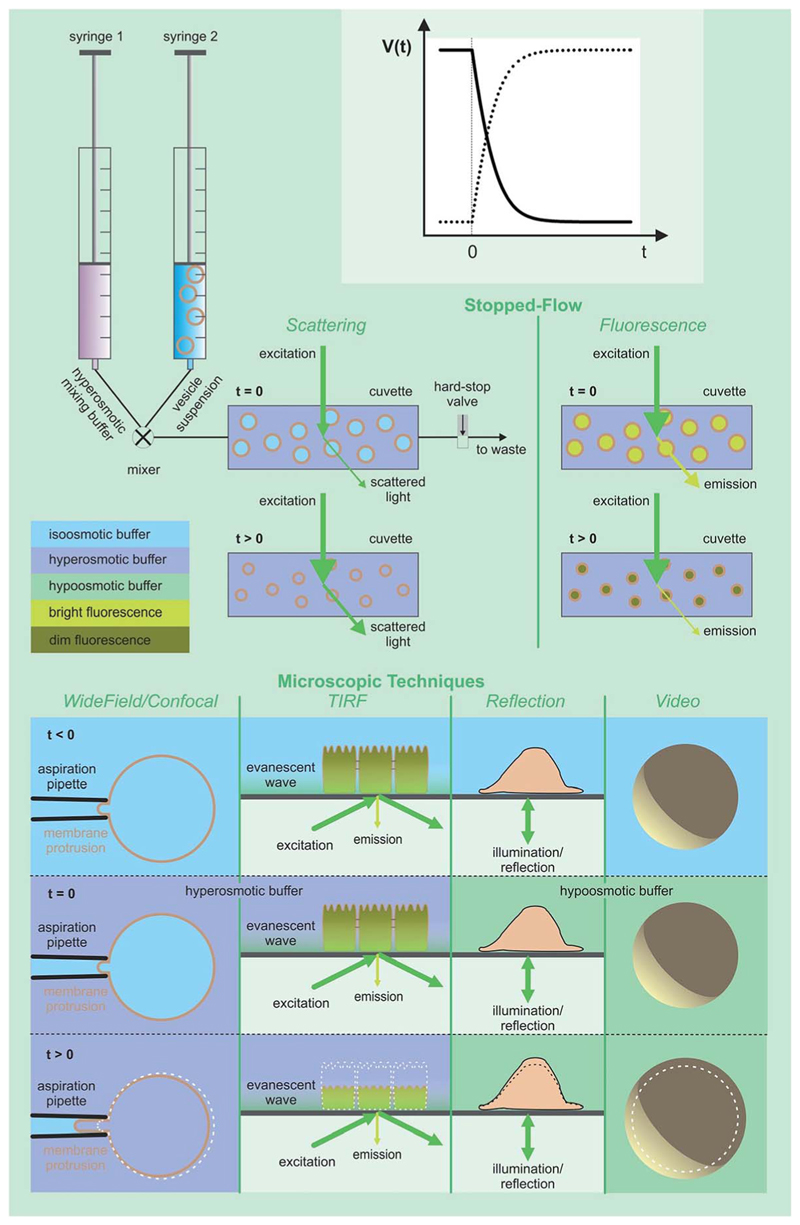
Assessment of volume changes inflicted by an osmotic challenge. The volume V(t) of LUVs and erythrocytes can be derived from the light scattering intensity or the fluorescence of a dye that is encapsulated in self-quenching concentrations. The rapid change in the external osmolyte concentration is achieved in a stopped-flow device (see main text for details). The *P*_f_ value of giant unilamellar vesicles (GUVs) can be obtained by using the microaspiration technique.^72,73^ GUV deflation lengthens the lipid projection length inside the aspiration pipette. The method is sensitive to small osmotic gradients. Alternatively, water efflux can be monitored by measuring the contact area of the surface adhered GUVs during deswelling.^74^ *V*(*t*) of adherent cells can either be studied *via* (i) laser scanning reflection microscopy,^75,76^ (ii) the dilution of an encapsulated dye in total internal reflection microscopy (TIRFM) mode,^77^ or (iii) laser scanning confocal microscopy.^78^ Oocyte swelling rates can be tracked by video microscopy.^79,80^ However, extensive internal unstirred layers^81^ as well as the resistance of the oolemma to unfolding^36^ may lead to an underestimation of *P*_f_.

Here we focus on the use of proteoliposomes (PLs), *i.e.* reconstituted large unilamellar vesicles (LUVs), for *P*_f_ measurements (Fig. 7 (top)). PLs are osmotically challenged and the resulting decrease in vesicular volume is derived by monitoring the intensity of the scattered light^82,83^ or by measuring the fluorescence of an encapsulated dye in self-quenching concentrations.^84,85^

A variety of approaches exist to extract *P*_f_ from *V*(*t*):

(α) First, numerically the differential-algebraic equations eqn (11) and (12) are solved for numerous *P*_f_ values. In a second step, the theoretical curves are fitted by a monoexponential function to find the characteristic time constants *τ*_c_ for vesicle deflation. Third, the recorded *V*(*t*) data are subjected to the same fitting procedure and the experimental constant *τ* for vesicle deflation is determined. Matching *τ* to the nearest *τ*_c_ value serves to identify *P*_f_ in a fourth step.^85^
(β) *P*_f_ is directly calculated from *τ*
  (13) $$P_{\text{f}}=\frac{r_{0}}{3V_{\text{w}}\tau }\times ∆\Pi$$
  where *r*_0_ is the initial vesicle radius. Depending on different models, Δ*Π* is equal to (a)^86^ *c*_out_^−1^, (b)^87^ (*c*_out_ − *c*_in,0_)^−1^ or (c)^88^ *c*_in,0_ *c*_out_^−2^.
(γ) The analytical solution of eqn (11) is used:^22^
  (14) $$V\left(t\right)=V_{0}\frac{c_{\text{in},0}}{c_{\text{out}}}\left\{1+L\left(\frac{c_{∆}}{c_{\mathrm{in},0}}\text{exp}\left(\frac{c_{∆}}{c_{\mathrm{in},0}}-\frac{AP_{\text{f}}V_{\text{w}}{c_{\text{out}}}^{2}}{V_{0}c_{\mathrm{in},0}}t\right)\right)\right\}$$
  where *c*_Δ_, and *L* are the increment in external osmolyte concentration (*c*_Δ_ = *c*_out_ − *c*_in,0_) and the Lambert function, defined by *L*(*x*)e^*L*(*x*)^ = *x*, respectively.

The approximate solutions (β) may lead to a large error in *P*_f_ – more than an order of magnitude in size.^89^ Moreover, *P*_f_ erroneously seems to depend on the osmotic gradient. In contrast, scanning electrochemical microscopy^58^ and fluorescence self-quenching experiments (analyzed using a numerical solution)^90^ show that *P*_f_ does not depend on the osmotic gradient. Eqn (14) has been used to calculate the *p*_f_ values of several AQPs,^22,37^ hSGLT1,^36^ and KcsA.^91^ It may be substituted by the following approximation that ensures acceptable accuracy:^89^

(15) $$P_{\text{f}}=\frac{r_{0}}{3V_{\text{w}}\tau }\times \frac{c_{\text{in},0}+c_{\text{out}}}{2{c_{\text{out}}}^{2}}.$$

Protein reconstitution is likely to result in two different vesicle populations: bare lipid vesicles and PLs.^92^ Consequently, two *P*_f_ values and two *V*(*t*) distributions have to be considered.^22,89^ Adapting the Rayleigh-Gans-Debye relation and comprising the change in size and refractive index of the lipid vesicles revealed a second order dependency with coefficients *a, b, d*:^22^

(16) $$I\left(t\right)=a+b\times V\left(t\right)+d\times V^{2}\left(t\right).$$

In contrast, eqn (15) assumes a linear dependence of *V*(*t*) on *I*(*t*), which, in the relevant range of *r*_0_ (~30 to 100 nm), leads to an acceptable accuracy. *r*_0_ has to be known with great accuracy as the error in *P*_f_ linearly depends on the ratio of assumed to real vesicle radii.^89^

### γ Protein counting

Accurate methods of transmembrane protein density estimations are based on direct visualization of single proteins in electrophysiology,^39,53,57,58^ EM,^40^ atomic force microscopy (AFM),^22^ or FCS.^22,36,37,69,93^ Even though the latter method relies on fluorescence labeling of the channel, FCS is a very convenient, reliable and time efficient method which can be used *in vivo* as well as *in vitro*. The protein concentration in the plasma membrane of polarized or non-polarized cells, GUVs or free standing planar lipid bilayers can be directly assessed. LUVs are too small to directly count the number of reconstituted proteins. Instead, two-step procedures are required. First the number of PLs is determined in the protein channel and second the vesicles are dissolved by detergent and the number of fluorescent micelles is counted in the focal volume.^22,36,37,69,93^ Depending on the protein, the addition of a mild detergent will lead to the formation of protein oligomers containing micelles and further addition of a harsh detergent leads to the formation of protein monomers containing micelles. In the ideal case, the ratio of fluorescent particles in the protein channel leads to an average number *n* of protein oligomers per proteoliposome, the number of protomers per oligomer, and the ratio of bare lipid vesicles to PLs. For the procedure to work, every protomer must bear a label. If the labeling efficiency is insufficient, the number of protomers per oligomer cannot be accurately determined. The reliability of this protein counting assay was verified by counting the channel density with AFM after spreading AQP-containing PLs on a solid support^94^ or on a cushion-supported membrane.^95^ Comparing the channel densities from AFM and FCS measurements revealed satisfactory agreement between the two complementary methods.^22^

## 7 Water flow through AQPs

Extending the dependence of *P*_f_(*N*) (Fig. 3) to longer channels required us to include a totally different class of proteins: AQPs. We first focused on human AQP1 (ref. 52 and 76) that is present in blood cells and in kidneys, on AQPZ,^96^ and on the aqua-glycerol facilitator GlpF,^56^ both from *E. coli.* In contrast to the water selective AQPZ, GlpF facilitates glycerol transport in addition to water.^97^ Channel reconstitution into liposomes at different protein to lipid ratios, followed by exact measurements of protein abundance using both FCS and high-speed-AFM, and the subsequent assessment of water efflux from osmotically challenged LUVs, allowed the total vesicle membrane permeability *P*_f_ to be plotted as a function of the membrane protein concentration.^22^ The slopes of these dependencies are indicative for *p*_f_.

**Fig. 8:**
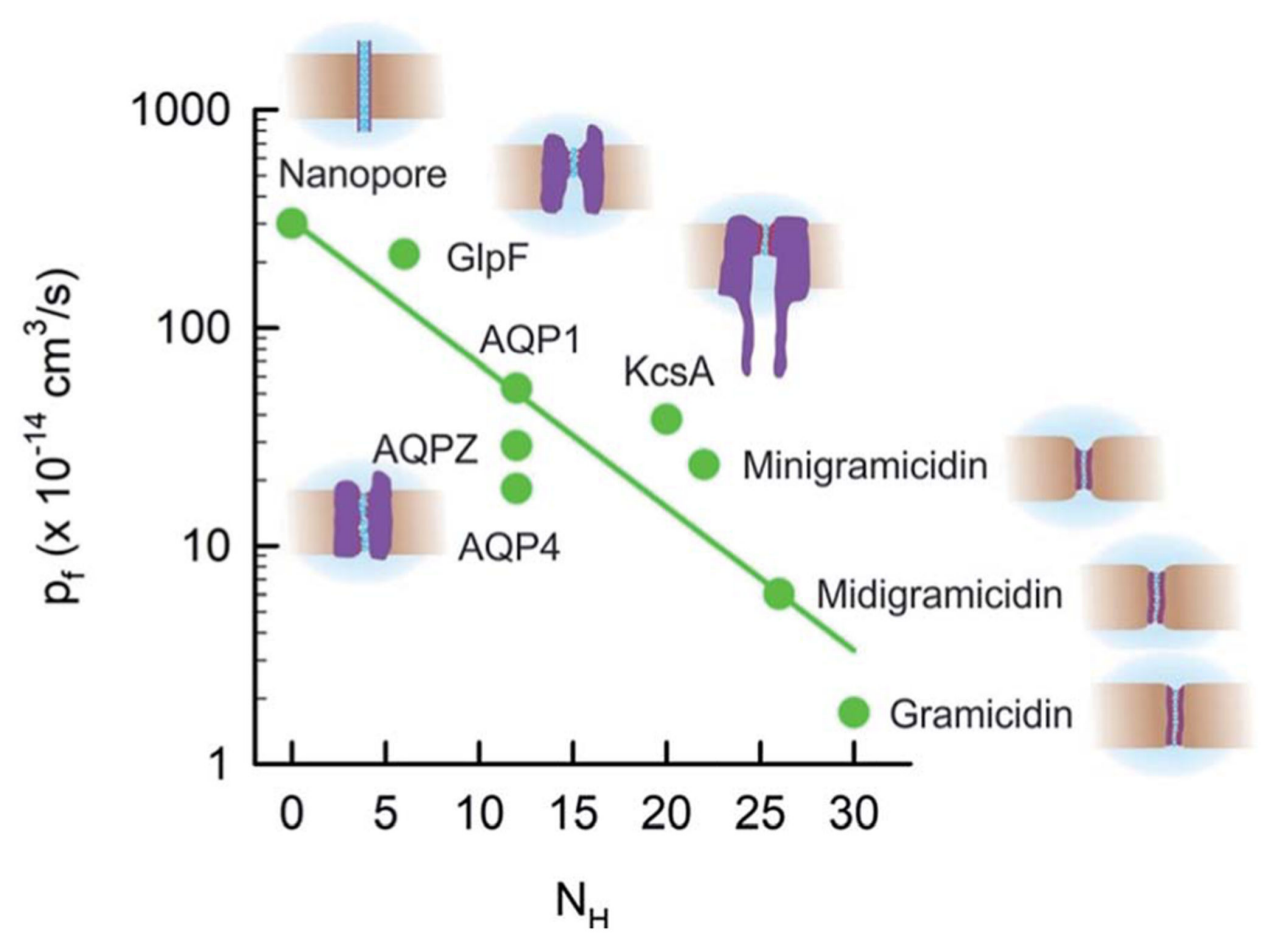
The unitary water channel conductance *p*_f_ depends exponentially on the number *N*_H_ of available hydrogen bond donors and acceptors. The data for AQP4 have been taken from ref. 37 and all other data are from ref. 22.

Although the pores of both AQPZ and AQP1 accommodate a long single-file that consists of eight water molecules, the *p*_f_ value of AQP1 exceeds the one of the much shorter KcsA. This observation clearly shows that *N* cannot generally serve as a determinant of *p*_f_. Obviously, the exponential dependence of *p*_f_ on *N* is not due to hopping of the water chain along *N* well-separated binding sites. The simple reason is that the number of water binding sites in both gramicidin or KcsA exceeds *N*. There are so many hydrogen bond forming residues in the walls of the single-file regions in both types of channels that the distance between two sets of different hydrogen bonding patterns is smaller than the diameter of a water molecule. In contrast, long stretches of the AQP pore contain only a few residues that are able to act as hydrogen bond donors or acceptors.^22^ That is, the total density of these residues is very different in the three types of channels. Plotting *p*_f_ as a function of the number *N*_H_ of potential hydrogen bond donors and acceptors in the walls of the single-file region revealed a logarithmic dependence (Fig. 8). The plot also includes GlpF, which has a shorter single-file region than AQP1 and thus its *N*_H_ value is also smaller. Accordingly, GlpF turned out to be the most efficient water facilitator (Fig. 2). Water molecules retain their bulk-like mobility within it. The *p*_f_ value of GlpF is only topped by nanotubes^25,98^ – possibly because carbon nanotubes are unable to form any hydrogen bonds within the pore. It is important to note that the *p*_f_ value for carbon nanotubes has been obtained *in silico* and is not yet confirmed experimentally.^99^

The exponential dependence of *p*_f_ on *N*_H_ (Fig. 8) predicts that the *p*_f_ value of carbon nanotubes does not depend on the tube length because *N*_H_ = 0 remains invariant. Indeed, the *p*_f_ value of single-file carbon nanotubes was found *in silico* to depend very weakly on *L*.^100^ For example, tubes that accommodate 17 instead of 3 water molecules have only a 20% lower *p*_f_ value.

Strikingly, the same invariance of *p*_f_ on *L* was obtained in molecular dynamics simulations that used a set of d, l polyalanine peptides with a typical β-helix gramicidin-A folding motif.^23^ Instead of being governed by hydrogen bond dynamics, water movement was limited by the dehydration penalty at the channel mouth. The reason for the discrepancy between the *in vitro* and *in silico* experiments is not entirely clear. A subsequent study identified transient channel blockages by the head groups of adjacent lipids as being responsible for the large quantitative differences in the *p*_f_ values of the *in silico* and *in vitro* studies.^101^

Fig. 8 clearly shows that *N*_H_ cannot be the only determinant of water flow through single-file channels. The three water channels – AQP4, AQP1, and AQPZ – offer the same number of hydrogen bonding residues within the water pathway – and yet their *p*_f_ values significantly differ from each other. An accompanying paper in this volume of *Faraday Discussions* proposes that the difference in *p*_f_ is due to the dehydration penalty that the water molecules face upon entering the single-file region:^37^ if only the hydrogen bonds to the preceding and following water molecules remain intact, the water molecules lose two of their four hydrogen bonds that they form in bulk. The energetic penalty of this process depends on the local environment at the channel mouth. Since positively charged residues are weaker hydrated than negatively charged amino acid side chains,^102,103^ they may contribute to a *p*_f_ increase. This hypothesis has been confirmed experimentally: the slowest AQP in Fig. 8, AQP4, offers the smallest number of positive charges. AQP1 has more than twice as many positively charged residues placed at the channel mouths and, accordingly, facilitates water transport three times faster.

**Fig. 9:**
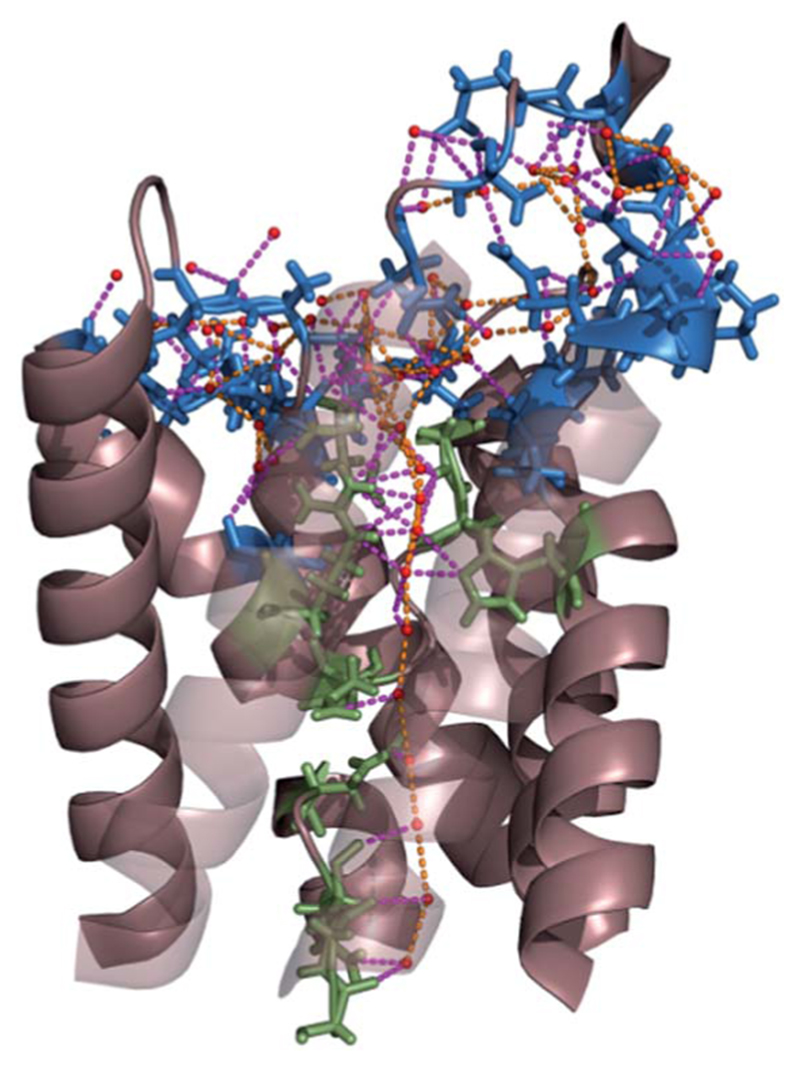
Network of hydrogen bonds within the single-file region of AQP and at the channel mouth. The residues that interact with single-file water molecules and with water molecules in the entrance region are depicted in green and blue, respectively. The H-bonds between water molecules and H-bonds between permeating water molecules and channel residues are shown in orange and pink, respectively. For clarity, the two front helices of yeast AQP1 [Protein Data Bank (PDB) #3Z0J] appear transparent.

We conclude that the network of hydrogen bonds at the channel mouth is important for the fine-tuning of *p*_f_ (Fig. 9). That is, hydrogen bonds in the single-file region are responsible for a variability in *p*_f_ that extends over 2 to 3 orders of magnitude, whereas the hydrogen bonded network in the channel mouth alters *p*_f_ two to three fold.

## 8 Energetics of water transport

H_2_O crosses lipid bilayer membranes by a solubility diffusion mechanism.^104^ Accordingly, a Gibbs activation energy barrier, $∆G_{\text{l}}^{‡}$, of 10.5 to 12.4 kcal mol^−1^ can be estimated for lipid membranes from two parameters of bulk hydrocarbons: 7.8 to 8.9 kcal mol^−1^ for the enthalpy of the partition coefficient and 2.6 to 3.4 kcal mol^−1^ for the activation of water diffusion.^105^ Some fine-tuning was achieved 40 years later by quantifying the impact of two more determinants of water diffusion across cholesterol-free bilayers:^82^ the area per lipid and the hydrocarbon thickness.^84,106^

The link between $∆G_{\text{l}}^{‡}$ and membrane permeability appears to be very robust – it also works for cholesterol-containing membranes. To obtain $∆G_{t}^{‡}$, *P*_f_ is measured at different temperatures and ln(*P*_f_) is plotted over 1/*T*. Cholesterol insertion into pure diphytanoyl phosphatidyl membranes increased $∆G_{\text{l}}^{‡}$ from 12 to 17–18 kcal mol^−1^ and reduced *P*_f_ to less than half of its initial value (at 30 °C).^107^ Molecular dynamics simulations exploit the link between permeability and $∆G_{\text{l}}^{‡}$ to accurately predict the membrane permeability of water and other small solutes from (i) local partition coefficients and (ii) local diffusion coefficients.^108^

Similarly, the Gibbs activation energy barrier $∆G_{t}^{‡}$ for facilitated water transport is intricately linked to *p*_f_. Two scenarios can be distinguished:^105^

(α) Water inside the pores has essentially bulk properties. In this case, the effective energy barrier for permeation is the activation energy for the selfdiffusion of water, about 4.6 kcal mol^−1^.^109^
(β) The value of 4.6 kcal mol^−1^ can be taken as a lower limit if water does not retain bulk properties inside the pore. The upper limit has been estimated to be about 15 kcal mol^−1^, assuming that in addition to the transport activation energy (4.6 kcal mol^−1^), the energy necessary for a water molecule to pass out of the solution and into the pore has to be considered. It is equal to 10.5 kcal mol^−1^ – the enthalpy of vaporization.^105^

Transition state theory allows a closer view on the link between *p*_f_ and $∆G_{t}^{‡}$. We first introduce the “hopping rate” *r* with which the water chain moves forward or backward:^25^

(17) $$r=p_{\text{f}}v_{\text{w}}.$$

Second we link *r* to $∆G_{t}^{‡}$:

(18) $$r=v_{0\;}\;\exp\left(-∆G_{t}^{‡}/k_{\text{B}}T\right)$$

where *ν*_0_ ≈ 10^13^ s^−1^ is the universal transition state theory attempt frequency. Eqn (17) and (18) allow calculation of *p*_f_ as:^99^

(19) $$p_{\text{f}}=v_{0}v_{\text{w}}\;\exp\left(-∆G_{t}^{‡}/k_{\text{B}}T\right).$$

**Fig. 10:**
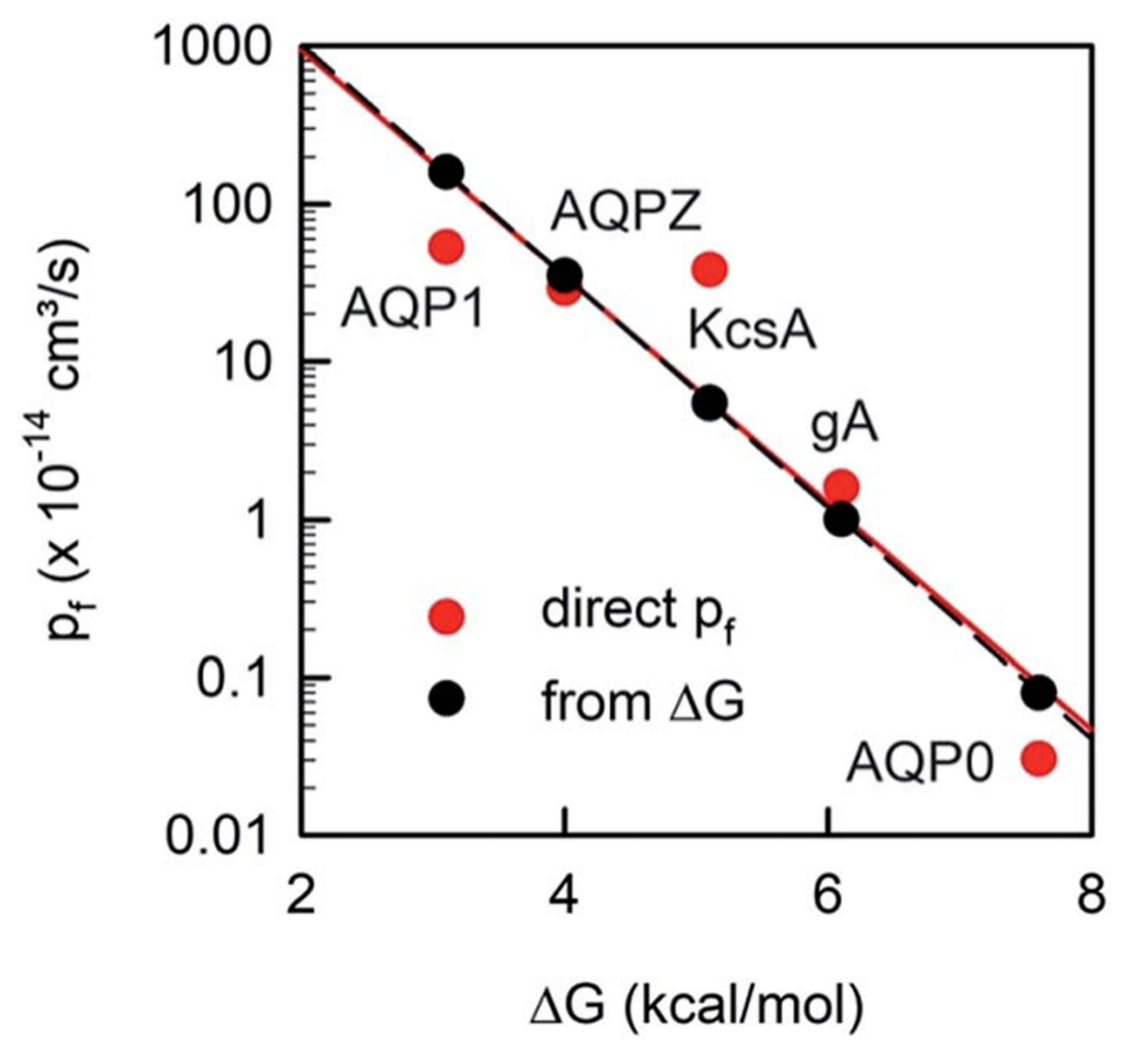
The unitary water permeability *p*_f_ and the Gibbs activation energy barrier $∆G_{t}^{‡}$ are intricately linked. The red dots represent experimental results: $∆G_{t}^{‡}$ has been reported for AQP1,^85^ AQPZ,^96^ KcsA,^58^ gA,^110^ and AQP0.^111^ *p*_f_ has also been obtained for AQP1,^22^ AQPZ,^22^ KcsA,^22^ gA,^45^ and AQP0.^40^ The black dots are calculated according to eqn (19).

Plotting the experimentally obtained *p*_f_ values that have been obtained by accurately counting the number of reconstituted channels for which the corre-sponding $∆G_{t}^{‡}$ values are known (Fig. 10), we find a satisfactory agreement between the prediction made by eqn (19) and the experiments.

Artificial water channels that could mimic the selectivity and extraordinarily fast flow possible in aquaporins would have a wide range of potential applications.^112^ Molecular dynamics simulations suggest that this goal is achievable: 8.1 Å wide nanotubes conduct water in single-file, while hydrated sodium and chloride ions are too large to pass *in silico*.^98,100^

In contrast, a recently published experimental study reports not only water, but also K^+^ and C^−^ transport across 10 nm long nanotubes with a pore diameter of 0.68 nm.^113^ The single nanotube conductivity at 100 mM K^+^ amounted to ~30 pS, which is comparable to that of potassium selective channels like Shaker^114^ or Kir2.1.^115^ How the nanotube achieves such efficiency without electrostatically attracting K^+^ to internal binding sites is unclear. Since surrogates for the water molecules of hydration of K^+^ are missing, the fully hydrated ion should be conducted. If this was the case, water molecules should be able to overtake each other in the channel, and yet H_2_O is believed to cross the tube in single-file.

At pH = 7.8, $∆G_{t}^{‡}$ for water transport across these tubes is reported to be equal to 24.1 kcal mol^−1^.^113^ Eqn (19) predicts an immeasurable small *p*_f_ value in the order of 10^−28^ cm^3^ s^−1^ and yet the authors claim *p*_f_ = 6.8 10^−13^ cm^3^ s^−1^. Such a disentanglement between the rate of a process and its activation energy is impossible because it violates fundamental thermodynamic laws.^99^ Moreover, the measured $∆G_{t}^{‡}$ value exceeds the upper limit of water transport through a pore of ~15 kcal mol^−1^ (see the preceding section). In their rebuttal in Science^116^ as well as in their contribution in the current *Faraday Discussions* volume,^117^ the authors do not provide evidence that water or ions pass the nanotubes. On the contrary, the inhibitory effect of calcium on both the ion and water fluxes is compatible with the view that calcium binding to the lipid alters lipid packing, which may partly seal the nanotube induced leak for water and ions in the bilayer.

## 9 Additional factors that affect *p*_f_

The example of AQP0 shows that factors besides the number of hydrogen bonds and surface charges may have an effect on *p*_f_: AQP0 offers 16 residues that accept or donate hydrogen bonds in its pore forming wall,^22^ and yet the *p*_f_ value is orders of magnitude smaller than that suggested by Fig. 8. Conformational transitions of the protein may explain the discrepancy. AQP0 was found in a closed^118^ as well as an open state.^119^ In addition, AQP0 was reported to be modulated by pH^120^ and calmodulin binding.^121^ Hence, the low *p*_f_ value of AQP0 (Fig. 2) may be due to a considerable amount of time the channel resides in its closed state.

The crystal structure of AqpZ also revealed two distinct conformations – different positions of Arg-189,^122^ yet its *p*_f_ value corresponds very well to the prediction that can be made from *N*_H_. The explanation is provided by recent NMR spectroscopy experiments. They revealed no indication of R189 gating in reconstituted AQPZ.^123^

Geometry effects may also play a minor role in fluid flow across biological channels and are believed to be important determinants for flow across nanotubes.^124^ A theoretical analysis of water transport through AQPs suggested that the form of the vestibule may boost AQP’s *p*_f_ value by several-fold. The *p*_f_ value was lowest for the channels with a cylindrical geometry, and highest for the channels with an hourglass shape.^24^ However, the geometry effects were derived from a continuum description that was unable to capture the water movement inside the central constriction zone. Contrary to the intuitive assumption that resistance is highest in the narrowest part of the channel, permeation through this one-water-molecule-wide part of the AQP pore was thought to occur free of any resistance.^24^ Experimental studies that would confirm this *in silico* prediction are still missing.

## Conclusions

We conclude that the major determinant of water flow across biological channels is the number of hydrogen bonds that water molecules may form on their way through the channel. Additional, but much less important factors, are: positive surface charges at the channel mouth, the presence of a closed conformational state of the channel, and possibly also the geometry of the vestibule at the channel mouth.
